# Effects of sowing date on photosynthetic characteristics, chlorophyll fluorescence and yield of different *Echium plantagineum* L. cultivars

**DOI:** 10.1038/s41598-023-38023-x

**Published:** 2023-09-04

**Authors:** Wu Wang, Longxue Wei, Hongming Li, Huifeng Xu, Zhen Xu, Chengming Yan, Ying Wu, Shengzhen Ji, Tao Wang

**Affiliations:** 1 Agricultural College of Jilin Agricultural University, No. 2888, Xincheng Street, Changchun, 130118 Jilin China; 2 Dezhou agricultural science research institute, No. 926, dexing zhong road, Dezhou, 253000 Shandong China; 3 Jilin Academy of Agricultural Sciences, No. 1363, Ecological Street, Changchun, 130033 Jilin China

**Keywords:** Plant sciences, Photosynthesis

## Abstract

The seed oil of *Echium plantagineum* L. is rich in unsaturated fatty acids. With the gradual development of the value of echium oil in food, medical care and cosmetics, the corresponding market demand has also increased. The selection of suitable cultivars and the increase of yield per unit area has also become one of the main objectives of current breeding and cultivation of *E. plantagineum*. To effectively use the local photothermal resources, to improve the use of light energy by *E. plantagineum*, and to enhance the growth and yield of *E. plantagineum*. *E. plantagineum* cultivars Blue Bedder and Mixed Bedding were used as research subjects to study the effects of different sowing dates (1 May, 8 May, 15 May, 22 May and 29 May) on the photosynthetic characteristics and yield of *E. plantagineum*. Under the same cultivar conditions, with the delay in sowing date, the leaf chlorophyll content (SPAD), photosynthetic rate (P_n_), transpiration rate (T_r_), stomatal limitation value (L_s_), photochemical quenching (qP), electron transfer rate (ETR), actual photochemical efficiency (ΦpsII) and yield of Blue Bedder decreased and reached a maximum at T1, while the SPAD, P_n_, T_r_, water use efficiency (WUE), L_s_, initial fluorescence (F_o_), maximum fluorescence (F_m_), qP, ETR, ΦpsII and yield of Mixed Bedding reached the maximum at T4. Blue Bedder should be sown early at T1 and Mixed Bedding late at T4 during planting, which will help to improve the photosynthetic characteristics and grain yield of *E. plantagineum*.

## Introduction

*Echium plantagineum* L. is a species of the genus *Echium* of the family *Boraginaceae*^1^. It is now widespread in the Mediterranean mainland. It is also found in Australia, New Zealand, South Africa, southern South America and the western United States^2^. The seeds of *E. plantagineum* contain highly polyunsaturated oil (approximately 14% linoleic acid, 10% γ-linolenic acid, 33% α-linolenic acid and 14% stearidonic acid), and almost half of this fatty acid is omega-3 fatty acid which cannot be synthesised by the human body^3^. Omega-3 long chain polyunsaturated fatty acids have certain beneficial effects on inflammatory and autoimmune diseases such as atherosclerosis, cancer, rheumatoid arthritis, asthma, and Alzheimer’s disease^4^. The United States Food and Drug Administration approved the use of *E. plantagineum* seed oil as a dietary ingredient in 2002, while the European Union classified it as a new type of food in 2008, allowing it to be added to various types of food^5^. As the value of echium oil in food, medical, and cosmetic products has been gradually developed, the corresponding market demand has increased^6^.

Photosynthetic efficiency plays an extremely important role in the yield formation of *E. plantagineum*. It not only affects the vegetative and reproductive growth of *E. plantagineum*, but also determines the filling rate and dry matter accumulation at the late grain stage. Therefore, understanding and improving the photosynthetic efficiency of the plant was of great importance for increasing the productivity and grain yield of *E. plantagineum*. Different sowing dates not only cause differences in ecological conditions such as photothermal resources during plant growth and development, but also alter photosynthesis and nutrient distribution during growth and development, thus affecting plant dry matter accumulation and yield^7^. Therefore, in order to make more effective use of local photothermal resources, improve light energy utilisation by *E. plantagineum*, and promote its growth and yield, it is of great practical significance to further strengthen the study of the effect of sowing date on the photosynthetic physiology and ecology of *E. plantagineum*. 90–95% of plant dry matter is derived directly or indirectly from photosynthesis^8^. Canopy light interception is the basis of plant photosynthesis. Canopy light interception and transmittance are important determinants of crop dry matter accumulation and subsequent yield^9^. Chlorophyll is the material basis of plant photosynthesis, and its content is closely related to the photosynthetic efficiency of crops^10^. The kinetic characteristic of chlorophyll fluorescence can directly reflect the photosynthetic performance of plants, and compared with the traditional “apparent” gas exchange index, the chlorophyll fluorescence parameters can better reflect the “intrinsic” characteristics of plant photosynthesis and can quickly, sensitively and non-destructively analyse the potential mechanism of environmental factor on photosynthesis^11, 12^. Photosynthesis is the material basis for the formation of biological yield and economic yield of plants, and improving the photosynthetic characteristics of plants is an important aspect to fully exploit the yield of plants^13^.

At present, the research on *E. plantagineum* mainly focuses on the development of its molecular substances and co-products such as honey, bee pollen, seed oil, shikonin and so on^6^, but there is little research on its cultivar. Different sowing dates will result in different environmental factors around the crop. By changing the sowing date, the utilisation rate and competition for natural climatic resources such as gas, water, heat and light of *E. plantagineum* can be adjusted, and its photosynthetic performance or yield composition can be improved to some extent, thus increasing its yield. However, the plant characteristics of different varieties of *E. plantagineum* are different. Therefore, regulating environmental factors by changing the sowing date has a good research prospect for improving flowering and fruiting and increasing seed yield of different varieties of *E. plantagineum*. In this experiment, the effects of sowing date on canopy light interception, chlorophyll content, photosynthesis, chlorophyll fluorescence and yield of *E. plantagineum*, in order to provide theoretical help and technical support for plantain production in this area.

## Materials and methods

### General situation of experimental site

**Figure 1:**
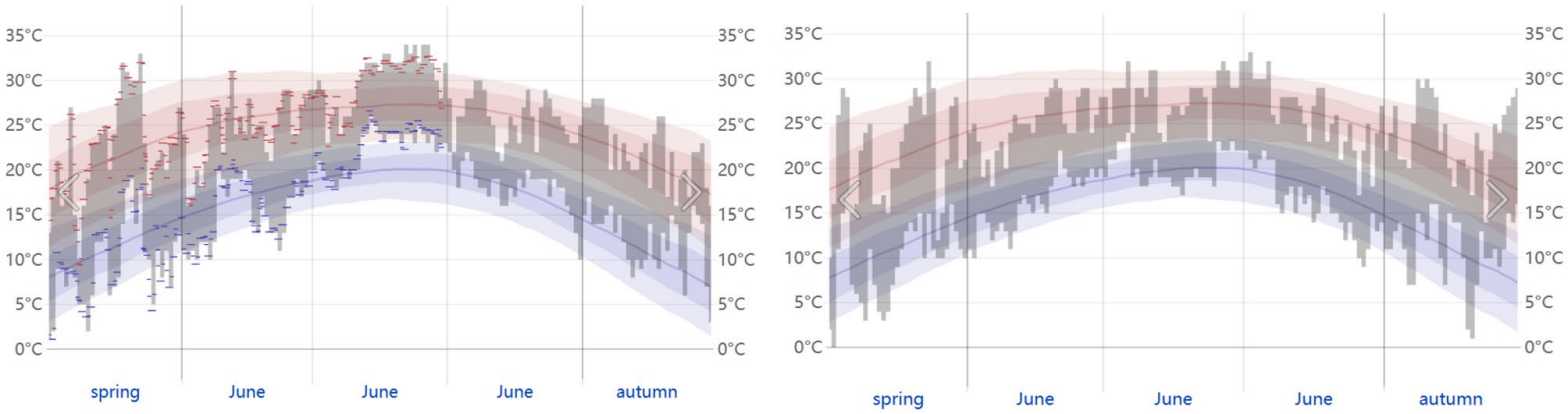
Historical temperature in growth period (2021 on the left and 2022 on the right). The daily range of reported temperatures (indicated by gray bars) and 24-h high (red checkmark) and low temperature (blue check) are placed above the daily average high (light red line) and low temperature (light blue line) and have 25–75% and 10–90% percentile bands. [Data quoted from: Weather Spark, website: https://zh.weatherspark.com].

The field trials were conducted in 2021 and 2022 in two growing seasons, and the experimental sites were located at the experimental base of Jilin Agricultural University in Changchun (43°53′N, 125°10′E), Jilin Province. The soil is a black loam with an obvious aggregate structure on the surface, rich in organic matter and high water-holding capacity, mainly containing montmorillonite and mica minerals. The basic fertility of the experimental field is as follows: organic matter 23.5 mg/kg, available phosphorus 24.3 mg/kg, available potassium 111.9 mg/kg, alkali-hydrolysable nitrogen 176.25 mg/kg, total nitrogen 1.645 g/kg, and soil pH value about 6.3. The temperature data in the experimental field of *E. plantagineum* during the whole growth period are shown in the following figures (the data corresponding to spring and autumn in the figure are in May and September, respectively). It can be seen that the phenomenon of short-term high temperatures often occurs in May in spring. Since the climatic difference during the growth period of two years was not significant, the experimental data of two years were averaged and analysed (Fig. 1).

### Experimental materials, design, and crop management

The seeds of *E. plantagineum* were obtained from a germplasm collection held at the School of Agriculture, Jilin Agricultural University, and the original collection was collected in England. Two cultivars of *E. plantagineum* were used in the study: Blue Bedder, which is characterised by slower growth and dense plant habit, and Mixed Bedding, which grows faster and has relatively few branches^14^. As there is little early spring rainfall in this area, soil moisture is insufficient before May, making it unsuitable for planting. In previous years, the local sowing date was around the beginning of May, so the sowing dates were split into 1 May (T1), 8 May (T2), 15 May (T3), 22 May (T4) and 29 May (T5). The experiment was designed using a random block of variety and sowing date, with a factor of 2*5, a total of 10 treatment groups, each treatment containing three replicates. Ridge width 65 cm, ridge height 10 cm. Each replicate consists of three ridges, each ridge is 5 m long, a corridor of 1 m is reserved, and the planting distance for hole sowing is 30 cm. Three for each acupoint. Test field peripheral protection row 1. Thinning is carried out after the seedlings have grown and one plant is reserved for each acupoint. Normal field management during the growing season. To ensure that the sowing date corresponds to the seed quality of *E. plantagineum*, the seeds of the lower third of the anterior flowers on the branches of the cyme are black (dry and fully mature), the seeds of the middle third are grey (physiologically mature), and the seeds of the upper third are green or the flowers are still mature for harvesting^15^.

### Measurement items and methods

The phenological period was subdivided and the criterion for subdivision was to reach more than 70% of the plants in each plot, and the corresponding indices were measured in each phenological period. Seedling stage: unfolding of the cotyledon to the eighth true leaf. Rosette stage: Eight true leaves spread out to the main stem/lateral branches for 2 cm. Extensional branching stage: Main stem/lateral branches extend 2 cm to 45% of the plant buds. Flowering stage: 45% of the buds are now harvested.

#### Canopy light interception

Clear and cloudless weather was selected during the extensional branching and flowering stages of *E. plantagineum*. Canopy photosynthetic active radiation (PAR) was measured at 11:30–14:30 using a canopy analyser. PAR interception rate (CaR) and transmittance (PeR) at different levels were calculated according to the following formula^16^.

$${\text{CaR}} = \left( {{\text{PAR}}_{{\text{n}}} {\text{ - PAR}}_{{\text{n - 1}}} } \right)/{\text{PAR}}_{{\text{T}}} \times 100\% ,$$

$${\text{PeR}} = {\text{PAR}}_{{{\text{n}} - {1}}} /{\text{PAR}}_{{\text{n}}} \times {1}00\% .$$

In the formula: PART denotes photosynthetic active radiation under natural light; N respectively denotes h (the height from the ground to the top of *E. plantagineum*) and 1/2 h, respectively; N-1 denotes 1/2 h and 0 h. When calculating the total CaR and total PeR of the canopy, n and n-1 indicated h and 0 h^9^.

#### Relative chlorophyll content

A hand-held SPAD-502 chlorophyll meter (manufactured in Japan)^17^ was used to the SPAD value of chlorophyll of *E. plantagineum* at seedling, rosette, branching and flowering stages. Three plants with uniform growth were selected from each plot, and the functional leaves with complete development in the middle canopy in a uniform light receiving direction were selected for measurement from each plant. The average of three measurement points on each leaf was taken, and the data were measured when the plants entered the corresponding phenology under the latest sowing date.

#### Photosynthetic characteristics

The photosynthetic rate (P_n_), stomatal conductance (G_s_), transpiration rate (T_r_), ambient CO_2_ concentration (C_a_) and intercellular CO_2_ concentration (C_i_) of the leaves were measured from 10:00 to 12:00 on a clear and windless day at the mid-flowering stage using a LI-6400 portable photosynthetic apparatus. The water use efficiency (WUE) was calculated as P_n_/T_r_^18^ and the stomatal limit value (L_s_) was calculated as 1-C_i_/C_a_^19^. For the determination, three plants of similar plant size were selected from each cell except for the edge, and the fully developed functional leaves in the middle canopy with the same light receiving direction were selected for each plant for the determination^20^.

#### Chlorophyll fluorescence

Chlorophyll fluorescence parameters of the leaves at the mid-flowering stage of *E. plantagineum* were measured using a model PAM-2500 fluorometer (WALZ, Germany). Three plants of similar size, except for the margin, and the fourth fully expanded branch and leaf (inverted four-leaf) on the upper part of the main stem were selected. Dark adaptation was performed for 20 min, followed by light adaptation. The fluorescence parameters measured included initial fluorescence (F_o_), maximum fluorescence (F_m_), maximum photochemical efficiency (F_v_/F_m_), actual photochemical chemical efficiency (ΦpsII), and electron transfer rate (ETR)^21^.

#### Yield

At the late stage of maturation, the complete plants of three *E. plantagineum* plants from each community were selected and brought back to the laboratory. The *E. plantagineum* seeds in the collected samples were dried in the sun, threshed and weighed to obtain the economic yield per plant and calculate the yield^22^. The seeds were then evenly mixed and three 500-grain weights were randomly selected to calculate the 1000-grain weight.

### Statistical analysis

Microsoft Excel2016 was used for basic data processing, spss was used for analysis of variance and correlation, and origin 2018 was used for mapping.

### Ethical approval

Experimental and field studies, including the collection of plant material (seeds), of *E. plantagineum* were conducted in accordance with relevant institutional, national and international guidelines and legislation. This experiment has been licensed for the collection of *E. plantagineum* and the corresponding scientific research.

## Results and analysis

### The plant canopy light interception

Photosynthetic Active Radiation (PAR) is the amount of solar radiation that can be used by green plants for photosynthesis. Under the same solar radiation conditions, the amount of photosynthetic active radiation (PAR) directly reflects the light absorption and utilisation of plants. CaR and PeR are important indicators of light interception in the crop canopy. As shown in Table 1, the total canopy CaR of the two E. plantagineum cultivars gradually increased with advancing growth period, and overall, the total canopy CaR of Blue Bedder was slightly higher than that of Mixed Bedding in each growth period, and the change in total canopy PeR was opposite. For Blue Bedder, the CaR of the total canopy in the extensional branching stage and that of the upper canopy in the flowering stage both tended to decrease with delay in sowing date, but the CaR of the total canopy in the flowering stage first increased and then decreased, and the interception rate reached its maximum at sowing date T2. The CaR and PeR of the lower canopy in the flowering stage of Blue Bedder were the lowest at T1 sowing date and the highest at T5 sowing date. Total canopy CaR and the CaR and PeR of the upper canopy in flowering stage of Blue Bedder were significantly different at T1 sowing time and T5 sowing dates, but the CaR and PeR of the lower canopy in flowering stage were not significantly different at each sowing date. There was no significant difference in the PeR of the lower canopy at flowering stage and the upper canopy at flowering stage among the treatments of each sowing date for Mixed Bedding. With the delay in sowing date, the CaR of total canopy of Mixed Bedding was T1 > T3 > T2 > T4 > T5 in the extensional branching stage, but it presented as T2 > T3 > T1 > T4 > T5 in the flowering stage, and the change in PeR was opposite to that of CaR. In the T2 and T3 sowing date treatments, the total canopy CaR of the Mixed Bedding at flowering stage was significantly different from that in the T5 treatment. From the above analysis, it could be concluded that delayed sowing would reduce the total canopy CaR of *E. plantagineum* to some extent and increase the total canopy PeR, and there was a certain difference in the total canopy CaR and PeR between the two cultivars. Sowing date and variety had significant effects on total canopy CaR and PeR, but their interaction had no significant effect.

**Table 1:** Effects of different treatments on interception rate and transmittance of photosynthetic active radiation in different canopy layers (2020–2022).

Treatments		Total canopy in extensional branching stage		Different layers of canopy in flowering stage				Total canopy at flowering stage	
				Upper layer		Lower layer			
Variety	Sowing date	CaR [%]	PeR [%]	CaR [%]	PeR [%]	CaR [%]	PeR [%]	CaR [%]	PeR [%]
Blue bedder	T1	92.03a	7.97e	83.11a	16.89b	14.45a	14.31a	97.56ab	2.44ef
	T2	91.38ab	8.62de	81.64ab	18.36ab	16.41a	14.65a	98.05a	1.95f.
	T3	86.35bc	13.65 cd	80.95ab	19.05ab	16.30a	15.35a	97.26abc	2.74def
	T4	83.58 cd	16.42bc	79.16ab	20.84ab	17.57a	15.80a	96.72abcd	3.28cdef
	T5	80.39d	19.61b	66.98ab	33.02a	27.49a	17.98a	94.47cde	5.53bcd
Mixed bedding	T1	84.42 cd	15.58bc	77.51ab	22.49ab	16.34a	27.16a	93.85de	6.15bc
	T2	83.20 cd	16.80bc	83.20a	16.80b	13.22a	23.98a	96.41abcd	3.59cdef
	T3	84.00 cd	16.00bc	79.30ab	20.70ab	15.59a	25.05a	94.89bcde	5.11bcde
	T4	79.83d	20.17b	71.63ab	28.37ab	21.01a	29.54a	92.64ef	7.36ab
	T5	73.26e	26.74a	65.83b	31.83ab	24.22a	34.43a	90.05f.	9.95a
Source of variance									
Variety		**	**	ns	ns	ns	**	**	**
Sowing date		**	**	*	*	ns	ns	**	**
Variety × sowing date		ns	ns	ns	ns	ns	ns	ns	ns

Small letters in the same column indicate significant difference (P < 0.05). *CaR* canopy light interception rate, *PeR* canopy light transmittance.

### The chlorophyll content (SPAD value)

Table 2 shows that the sowing date had a significant effect on the chlorophyll content of *E. plantagineum* at the seedling, rosette and extensional branching stages. Except for the flowering stage of Mixed Bedding, the chlorophyll content of *E. plantagineum* at different growth stages of the two cultivars gradually decreased with the delay in sowing date, reaching the maximum at sowing date T1 and the minimum at sowing date T5. In the flowering stage, the chlorophyll content of Mixed Bedding showed T1 < T3 < T2 < T5 < T4, and reached the maximum at T4 sowing date, which was significantly different from T1. The chlorophyll of Blue Bedder was significantly different from that of T4 and T5 at seedling, rosette, branching and flowering stages and at T1 sowing date. The chlorophyll of the T1 plant of Mixed Bedding was significantly different from those of T2, T3, T4 and T5 at the seedling and flowering stages. Therefore, it could be concluded that delayed sowing was not conducive to improving the chlorophyll content of plants of Blue Bedder and Mixed Bedding at any growth stage, but moderate late sowing of Mixed Bedding at T4 stage was conducive to improving the chlorophyll content of *E. plantagineum* at flowering stages. The variety had a significant effect on the chlorophyll at the rosette stage and the flowering stage, and the interaction of sowing date and variety had a significant effect on the chlorophyll content at the flowering stage of *E. plantagineum*.

**Table 2:** Effect of sowing date on chlorophyll content of different *E*. *plantagineum* varieties at different growth stages. (SPAD value) (2020–2022).

Variety	Sowing date	Seedling stage	Rosette stage	Extensional branching stage	Flowering stage
Blue bedder	T1	24.60 ± 0.40a	26.17 ± 0.75a	31.65 ± 0.74a	56.38 ± 0.41a
	T2	23.30 ± 0.72ab	25.03 ± 0.65ab	30.58 ± 0.60ab	53.50 ± 2.03b
	T3	21.70 ± 0.44bc	25.03 ± 0.09ab	30.40 ± 1.06ab	52.89 ± 0.89bc
	T4	20.83 ± 0.20c	22.80 ± 0.32 cd	29.00 ± 0.84bc	50.13 ± 1.15 cd
	T5	18.47 ± 0.57d	20.50 ± 0.59e	27.18 ± 0.39 cd	48.49 ± 0.92de
Mixed bedding	T1	24.67 ± 0.19a	26.20 ± 0.81a	30.78 ± 0.72ab	44.13 ± 0.40f.
	T2	22.60 ± 0.62b	25.83 ± 0.38ab	30.60 ± 0.60ab	47.49 ± 0.35de
	T3	22.50 ± 0.81b	25.17 ± 0.34ab	30.00 ± 0.42ab	46.49 ± 0.72ef
	T4	20.57 ± 0.32c	24.33 ± 0.29bc	29.65 ± 0.62ab	49.35 ± 0.91de
	T5	18.53 ± 0.62d	21.80 ± 0.52de	26.85 ± 0.29d	48.30 ± 0.85de
Source of variance					
Variety		ns	*	ns	**
Sowing date		**	**	**	ns
Variety × sowing date		ns	ns	ns	**

Values are means ± SD. Small letters in the same column indicate significant difference (P < 0.05).

### The leaf photosynthetic characteristics

As shown in Table 3, the differences in leaf T_r_ and WUE of the two varieties at each sowing date were not significant. With the delay in sowing date, leaf P_n_, T_r_ and L_s_ of Blue Bedder tended to decrease, while C_i_ and G_s_ tended to increase, reaching their maximum and minimum values respectively at sowing date T1. There were significant differences in P_n_, C_i_ and L_s_ of Blue Bedder with those of T1, T2 and T3 at sowing date T5. With the delay in sowing date, the leaf P_n_ and T_r_ of Mixed Bedding first increased and then decreased, then increased and then decreased, reaching the maximum at sowing date T4. At sowing date T1, there were significant differences in leaf P_n_, C_i_ and L_s_ of Mixed Bedding and those of T2, T3, T4 and T5. Therefore, it could be concluded that normal sowing of Blue Bedder and moderately late sowing of Mixed Bedding were conducive to improving the photosynthetic characteristics of the corresponding *E. plantagineum* at the flowering stage. In addition, sowing date had significant effects on leaf P_n_, C_i_ and L_s_, and there were significant differences in P_n_, C_i_, T_r_ and L_s_ between the two cultivars. The effect of cultivar on photosynthetic characteristics was greater than the difference at sowing.

**Table 3:** Effects of sowing date on photosynthetic characteristics of different *E*. *plantagineum* varieties at flowering stage. (2020–2022).

Variety	Sowing date	P_n_ (μmol m^–2^ s^–1^)	G_s_ (mol m^–2^ s^–1^)	C_i_ (μmol mol^–1^)	T_r_ (mmolm^–2^ s^–1^)	WUE (%)	L_s_
Blue bedder	T1	45.43 ± 0.32a	0.55 ± 0.03b	154.00 ± 6.56e	8.06 ± 0.08a	5.64 ± 0.04a	0.58 ± 0.02a
	T2	45.33 ± 0.26a	0.61 ± 0.01ab	162.33 ± 2.33de	7.96 ± 0.55ab	5.75 ± 0.41a	0.56 ± 0.00ab
	T3	44.33 ± 0.35b	0.64 ± 0.00ab	163.00 ± 2.89de	7.79 ± 0.25abc	5.70 ± 0.21a	0.55 ± 0.01ab
	T4	43.77 ± 0.19bc	0.65 ± 0.03ab	168.67 ± 4.33de	7.79 ± 0.15abc	5.63 ± 0.13a	0.53 ± 0.01bc
	T5	43.37 ± 0.26c	0.67 ± 0.06ab	188.00 ± 6.25bc	7.6 ± 0.17abc	5.71 ± 0.16a	0.49 ± 0.01de
Mixed bedding	T1	40.07 ± 0.34e	0.74 ± 0.05a	211.67 ± 4.70a	7.12 ± 0.30bc	5.65 ± 0.19a	0.44 ± 0.01f.
	T2	41.90 ± 0.26d	0.58 ± 0.01b	177.00 ± 0.58 cd	7.24 ± 0.16abc	5.79 ± 0.16a	0.52 ± 0.00bcd
	T3	41.37 ± 0.15d	0.58 ± 0.06b	195.33 ± 3.84b	7.03 ± 0.27c	5.91 ± 0.24a	0.48 ± 0.01e
	T4	42.13 ± 0.41d	0.64 ± 0.06ab	185.00 ± 7.51bc	7.75 ± 0.09abc	5.44 ± 0.08a	0.50 ± 0.02cde
	T5	41.77 ± 0.13d	0.58 ± 0.03b	194.00 ± 3.00b	7.41 ± 0.17abc	5.64 ± 0.13a	0.48 ± 0.01e
Source of variance							
Variety		**	ns	**	**	ns	**
Sowing date		*	ns	**	ns	ns	**
Variety × sowing date		**	*	**	ns	ns	**

Values are means ± SD. Small letters in the same column indicate significant difference (P < 0.05). P_n_ net photosynthetic rate, G_s_ stomatal conductance, C_i_ intercellular CO_2_ concentration, T_r_ transpiration rate, *WUE* water-use efficiency, L_s_ stomatal limitation.

### The chlorophyll fluorescence

As shown in Table 4, F_o_, F_v_/F_m_, qP, ETR and ΦpsII showed significant differences between the two varieties, and the sowing date had no significant effect on chlorophyll fluorescence parameters. The interaction between sowing date and varieties had significant effects on qP, and the influence of varieties on chlorophyll fluorescence parameters was greater than that of sowing date. F_v_/F_m_, ETR and ΦpsII of Blue Bedder were significantly higher than those of Mixed Bedding, while F_o_, F_m_, F_v_/F_m_, ETR and ΦpsII of Blue Bedder and Mixed Bedding did not differ significantly from those of their respective varieties at sowing date. With a delay in sowing date, F_o_ and F_m_ of Blue Bedder first increased and then decreased, and reaching a maximum at T3, while NPQ showed the opposite. QP, ETR and ΦpsII of Blue Bedder decreased with the sowing date, reaching their maximum values at sowing date T1. The F_o_, F_m_, qP, ΦpsII and ETR of Mixed Bedding initially increased, then decreased, then increased and then decreased with the delay in sowing date, reaching the maximum at sowing date T4, but the corresponding NPQ value was the minimum at that time.

**Table 4:** Effects of sowing date on chlorophyll fluorescence parameters of different *E*. *plantagineum* varieties at flowering stage (2020–2022).

Variety	Sowing date	F_o_	F_m_	F_v_/F_m_	qP	NPQ	ETR	ΦpsII
Blue bedder	T1	226.00 ± 10.58c	1254.67 ± 104.67a	0.82 ± 0.01a	0.35 ± 0.01a	1.57 ± 0.12a	118.24 ± 1.49a	0.19 ± 0.00a
	T2	244.67 ± 12.41c	1390.33 ± 118.90a	0.82 ± 0.01a	0.32 ± 0.02ab	1.41 ± 0.15ab	111.56 ± 4.02ab	0.18 ± 0.01ab
	T3	262.00 ± 14.36bc	1472.33 ± 77.80a	0.82 ± 0.00a	0.3 ± 0.01ab	1.22 ± 0.09ab	111.34 ± 3.78ab	0.17 ± 0.01ab
	T4	238.33 ± 14.17c	1325.33 ± 59.48a	0.82 ± 0.02a	0.3 ± 0.03ab	1.37 ± 0.02abc	104.87 ± 11.43abc	0.16 ± 0.02abc
	T5	226.33 ± 15.51c	1207.00 ± 95.11a	0.81 ± 0.02ab	0.29 ± 0.02ab	1.59 ± 0.16bcd	96.94 ± 8.42bcd	0.15 ± 0.01bcd
Mixed bedding	T1	290.67 ± 0.67bc	1277.67 ± 66.92a	0.77 ± 0.01bc	0.22 ± 0.01c	0.89 ± 0.30d	84.05 ± 1.74d	0.13 ± 0.00d
	T2	327.00 ± 39.43ab	1398.33 ± 67.92a	0.77 ± 0.02bc	0.29 ± 0.03ab	1.47 ± 0.11 cd	90.54 ± 1.91 cd	0.14 ± 0.00 cd
	T3	325.00 ± 30.09ab	1364.00 ± 72.50a	0.76 ± 0.01c	0.28 ± 0.02bc	1.59 ± 0.21d	85.84 ± 2.64d	0.13 ± 0.00d
	T4	386.67 ± 51.94a	1549.67 ± 209.31a	0.75 ± 0.01c	0.31 ± 0.02ab	1.23 ± 0.17bcd	95.21 ± 4.54bcd	0.15 ± 0.01bcd
	T5	328.67 ± 4.67ab	1392.67 ± 14.52a	0.76 ± 0.00c	0.29 ± 0.02ab	1.48 ± 0.13 cd	90.79 ± 4.90 cd	0.14 ± 0.01 cd
Source of variance								
Variety		**	ns	**	*	ns	**	**
Sowing date		ns	ns	ns	ns	ns	ns	ns
Variety × sowing date		ns	ns	ns	*	ns	ns	ns

Values are means ± SD. Small letters in the same column indicate significant difference (P < 0.05). F_o_ Initial fluorescence, F_m_ Maximum fluorescence, F_v_/F_m_ maximal quantum yield of PSII photochemistry, qP photochemical quenching coefficient, NPQ nonphotochemical quenching, ΦPSII actual photochemical efficiency of PSII, *ETR* electron transport rate.

### The grain yield

**Figure 2:**
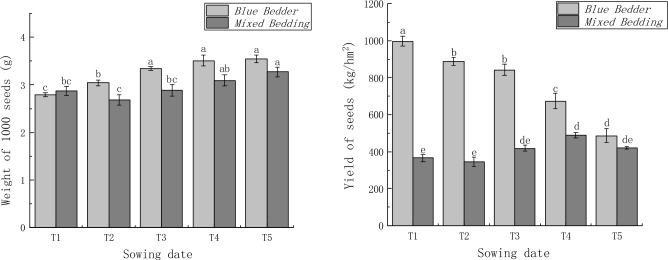
Effects of sowing date on 1000-grain weight and grain yield of different *E*. *plantagineum* Varieties (2020–2022). Histograms capped with different letters indicate significant difference (P < 0.05).

Table 5 shows that the variety and sowing date have significant effects on 1000 grain weight and grain yield, and the interaction between variety and sowing date has a significant effect on grain yield. From Fig. 2 it can be seen that the yield of Blue Bedder was significantly higher than that of Mixed Bedding at each sowing date. The thousand grain weight of Mixed Bedding at T1 was higher than that of Blue Bedder, and the thousand grain weight of Blue Bedder at other sowing dates was significantly higher than that of Mixed Bedding. As the sowing date was delayed, the 1000 grain weight of Blue Bedder increased and the yield decreased, reaching a maximum of 996.72 kg/hm^2^ at T1. The 1000 grain weight of Blue Bedder at sowing dates T3, T4 and T5 was significantly different from that at T1 and T2 treatments, while the yield at sowing date T1 was significantly different from that at T2, T3, T4 and T5 treatments. The delayed 1000 grain weight of Mixed Bedding first decreased and then increased with the sowing date, and the minimum 1000 grain weight was 2.68 g at sowing date T2. In addition, the grain yield of Mixed Bedding in the different sowing date treatments was T2 < T1 < T3 < T5 < T4, the minimum yield at T2 was 344.4 kg/hm^2^, and the maximum yield at T4 was 489.17 kg/hm^2^. The 1000 grain weight of Mixed Bedding at T5 was significantly different from T1, T2 and T3 treatments, while the yield at T4 was significantly different from T1 and T2 treatments. The maximum yield of Blue Bedder was significantly different from that of Mixed Bedding, and the maximum yield of Blue Bedder was 203.76% higher than that of Mixed Bedding. From the above analysis, it could be concluded that delaying the sowing date could significantly increase the 1000 grain weight of *E. plantagineum*, while Mixed Bedding at T4 sowing date and Blue Bedder at T1 sowing date contributed to the significant increase in grain yield, and Blue Bedder had yield advantages over Mixed Bedding.

**Table 5:** Analysis of variance of 1000-seeds weight and grain yield (F value) (2020–2022).

Source of variance	1000-seeds weight	Grain yield
Variety	25.346**	488.941**
Sowing date	16.345**	21.011**
Variety × sowing date	2.785	41.016**

** is significant at P ＜0.01.

### The correlation analysis

As shown in Table 6, the total canopy CaR, SPAD, P_n_, T_r_, L_s_, F_v_/F_m_, qP, ETR and ΦpsII of *E. plantagineum* at the flowering stage had a linear positive correlation with yield, while the total canopy PeR, C_i_ and F_o_ had a significant negative correlation with yield. The decision coefficients R^2^ of leaves SPAD, P_n_, C_i_, T_r_, L_s_, ETR and ΦpsII were greater than 0.7, which proved the improvement of the photosynthetic characteristics of the plant and had a positive effect on the improvement of the crop yield. Table 7 shows that the varieties had a significant positive correlation with 1000 grain weight and qP, an extremely significant positive correlation with yield, SPAD, P_n_, T_r_, L_s_, F_v_/F_m_, ETR, ΦpsII and total canopy CaR at the flowering stage, and an extremely significant negative correlation with C_i_, F_o_ and total canopy PeR at the flowering stage. Sowing date had a very significant positive correlation with 1000 grain weight and total canopy PeR at flowering stage, and a very significant negative correlation with total canopy CaR at flowering stage. Total canopy CaR at flowering stage had a very significant positive correlation with P_n_, L_s_, F_v_/F_m_, ETR, ΦpsII, SPAD and yield, a significant positive correlation with SPAD, a very significant negative correlation with C_i_, and a significant negative correlation with F_o_, while the correlation with the total canopy PeR at flowering stage was the opposite. This indicated that sowing date had a direct effect on canopy structure and thus indirectly affect photosynthesis, fluorescence characteristics and grain yield.

**Table 6:** Effects of photosynthetic characteristics and fluorescence characteristics of leaves at flowering stage on the yield of *E*. *plantagineum*.

Yield (y)		
Total canopy CaR	x_1_	y = 64.8x_1_–5575.9 (R^2^ = 0.4688**)
Total canopy PeR	x_2_	y = − 64.8x_2_ + 904.1 (R^2^ = 0.4688**)
SPAD	x_3_	y = 62.245x_3_–2502.1 (R^2^ = 9007**)
P_n_	x_4_	y = 124.12x–4738 (R^2^ = 0.8571**)
G_s_	x_5_	y = − 1226.9x + 1358 (R^2^ = 0.0829^ ns^)
C_i_	x_6_	y = − 11.476x_6_ + 2657 (R^2^ = 0.7546**)
T_r_	x_7_	y = 580.98x_7_–3808.5 (R^2^ = 0.7576**)
WUE	x_8_	y = − 198.46x_8_ + 1720.9 (R^2^ = 0.0104^ ns^)
L_s_	x_9_	y = 4903.8x_9_–1923.2 (R^2^ = 0.7791**)
F_o_	x_10_	y = − 2.8101x_10_ + 1394.8 (R^2^ = 0.4142**)
F_m_	x_11_	y = − 0.0938x_11_ + 720.29 (R^2^ = 0.0015^ ns^)
F_v_/F_m_	x_12_	y = 6424.8x_12_–4483.2 (R^2^ = 0.6602**)
qP	x_13_	y = 5267.5x_13_–961.5 (R^2^ = 0.5331**)
NPQ	x_14_	y = 168.86x_14_ + 359.05 (R^2^ = 0.024^ ns^)
ETR	x_15_	y = 19.549x_15_–1341.7 (R^2^ = 0.9458**)
ΦpsII	x_16_	y = 11191x_16_–1131.1 (R^2^ = 0.9381**)

* and ** are significantly correlation at P < 0.05 and P < 0.01, respectively.

*ns* is not significantly correlation. *CaR* canopy light interception rate, *PeR* canopy light transmittance, P_n_ net photosynthetic rate, G_s_ stomatal conductance, C_i_ intercellular CO_2_ concentration, T_r_ transpiration rate, *WUE* water-use efficiency, L_s_ stomatal limitation, F_o_ Initial fluorescence, F_m_ Maximum fluorescence, F_v_/F_m_ maximal quantum yield of PSII photochemistry, qP photochemical quenching coefficient, NPQ nonphotochemical quenching, ΦPSII actual photochemical efficiency of PSII, *ETR* electron transport rate.

**Table 7:** Correlation between sowing date and photosynthesis, chlorophyll fluorescence parameters and yield characteristics of *E. plantagineum* at flowering stage.

Correlation	Variety	Sowing date	Weight of a 1000 seeds	Yield	P_n_	G_s_	C_i_	T_r_	WUE	L_s_	F_o_	F_m_	F_v_/F_m_	qP	NPQ	ΦPSII	ETR	SPAD	Total canopy CaR	Total canopy PeR
Variety	1																			
Sowing date	0.000	1																		
Weight of a 1000 seeds	0.456*	0.719**	1																	
Yield	0.804**	− 0.301	0.090	1																
P_n_	0.864**	− 0.084	0.213	0.891**	1															
G_s_	0.003	0.032	0.221	− 0.195	− 0.222	1														
C_i_	− 0.692**	0.181	− 0.048	− 0.805**	− 0.870**	0.437*	1													
T_r_	0.534**	0.001	0.281	0.581**	0.577**	− 0.112	− 0.531**	1												
WUE	0.004	− 0.078	− 0.193	− 0.028	0.058	− 0.048	− 0.012	− 0.778**	1											
L_s_	0.636**	− 0.212	− 0.033	0.787**	0.860**	− 0.485**	− 0.987**	0.512**	0.031	1										
F_o_	− 0.737**	0.147	− 0.327	− 0.554**	− 0.566**	− 0.031	0.438*	− 0.310	− 0.040	− 0.380*	1									
F_m_	− 0.193	0.090	− 0.133	− 0.016	− 0.082	0.096	− 0.035	− 0.029	− 0.025	0.036	0.633**	1								
F_v_/F_m_	0.821**	− 0.132	0.304	0.733**	0.703**	0.103	− 0.615**	0.406*	0.025	0.543**	− 0.804**	− 0.057	1							
qP	0.384*	0.012	− 0.028	0.497**	0.652**	-0.415*	− 0.663**	0.437*	− 0.038	0.704**	− 0.194	− 0.111	0.209	1						
NPQ	0.163	0.218	0.073	0.117	0.281	-0.433*	− 0.250	0.215	− 0.044	0.271	− 0.194	− 0.141	0.176	0.564**	1					
ΦPSII	0.709**	− 0.162	0.127	0.799**	0.840**	-0.100	− 0.814**	0.412*	0.123	0.797**	− 0.574**	− 0.035	0.730**	0.683**	0.139	1				
ETR	0.709**	− 0.162	0.127	0.799**	0.840**	-0.100	− 0.814**	0.412*	0.123	0.797**	− 0.574**	− 0.035	0.730**	0.682**	0.029	1.000**	1			
SPAD	0.666**	− 0.164	0.110	0.828**	0.793**	-0.263	− 0.739**	0.695**	− 0.241	0.729**	− 0.313	0.044	0.493**	0.644**	0.258	0.696**	0.696**	1		
Total canopy CaR	0.601**	− 0.494**	− 0.110	0.587**	0.584**	0.000	− 0.608**	0.162	0.265	0.587**	− 0.383*	− 0.038	0.479**	0.276	− 0.050	0.512**	0.512**	0.367*	1	
Total canopy PeR	− 0.601**	0.494**	0.110	− 0.587**	− 0.584**	0.000	0.608**	− 0.162	− 0.265	− 0.587**	0.383*	0.038	− 0.479**	− 0.276	0.050	− 0.512**	− 0.512**	− 0.367*	− 1.000**	1

* and ** are significantly correlation at P < 0.05 and P < 0.01, respectively.

## Discussions

The yield potential of *E. plantagineum* depends on the amount of photosynthate accumulated during the growing season or on the proportion of the total biomass allocated to the grain at harvest. CaR and PeR in the canopy are important factors that determine the dry matter accumulation and subsequent crop yield^9^. The results of this study showed that under different sowing dates and varieties, the changing trends of CaR and PeR were opposite. As the growth period progressed, the total canopy CaR increased, and the upper canopy CaR at the flowering stage was much higher than that of the lower canopy, while the total canopy CaR of Blue Bedder was relatively higher than that of Mixed Bedding. This was consistent with the fact that Blue Bedder was more densely branched than Mixed Bedding^23^. The results showed that sowing date and variety had significant effects on total canopy CaR and PeR, but their interaction had no significant effect.

Chlorophyll is the material basis of photosynthesis in plants^24^. The results of this experiment showed that the chlorophyll content of the main functional leaves of *E. plantagineum* was the highest during the flowering stage. Except for the flowering stage of Mixed Bedding, the chlorophyll content of leaves of *E. plantagineum* at each growth stage decreased with the delay in sowing date. The chlorophyll value before late sowing is relatively low, mainly because the corresponding functional leaves are delayed in spreading. In this study, the chlorophyll of T1 plant of Mixed Bedding was significantly lower than that of T2, T3, T4 and T5 at flowering stage and the specific reason required further investigation. In addition, this study also found that the chlorophyll content of Blue Bedder and Mixed Bedding was significantly different at the flowering stage, while the sowing date had a significant effect on the chlorophyll content at the seedling, rosette and extensional branching stages. The interaction between the sowing date and cultivar had a significant effect on the chlorophyll content at the flowering stage.

Photosynthesis is the basis of plant dry matter accumulation and yield formation, and improving photosynthesis is of great importance for improving plant productivity and grain yield. Chlorophyll fluorescence can quickly, sensitively and non-invasively study and detect the true behaviour of photosynthesis of intact plants in different environments, and is widely used to evaluate the effects of photosynthetic apparatus function and environmental changes on it^25^. The results showed that sowing Blue Bedder at T1 was beneficial to increase the plant leaves P_n_, T_r_, L_s_, qP, ETR and ΦpsII while reducing C_i_ and G_s_, and sowing Mixed Bedding moderately later at T4 was beneficial to increase the leaves P_n_, T_r_, F_o_, F_m_, qP, ΦpsII and ETR. There were significant differences in P_n_, C_i_, T_r_, L_s_, F_o_, F_v_/F_m_, qP, ETR and ΦpsII between the two cultivars, while the P_n_, T_r_, F_v_/F_m_, ETR and ΦpsII at flowering stage of Blue Bedder were significantly higher than that of Mixed Bedding. It indicated that sowing Blue Bedder at T1 and sowing Mixed Bedding at T4 could improve the photosynthetic performance of plants, and the photosynthetic performance and fluorescence performance of Blue Bedder were significantly higher than that of Mixed Bedding. In addition, F_v_/F_m_ is the maximum primary photochemical quantum efficiency of PSII and an important parameter of photochemical reaction, which reflects the potential maximum photosynthetic capacity of plants^26^. F_v_/F_m_ of most higher plants ranged from 0.8 to 0.85, and when F_v_/F_m_ decreased, it represented that the plants were under stress^27–29^. In the experiment, the F_v_/F_m_ of Blue Bedder was in the range of 0.8–0.85, while the F_v_/F_m_ of Mixed Bedding was less than 0.8 in the range of 0.75–0.78, which could be due to the different responses of the two varieties to the same environmental change.

In this study, sowing date significantly affected the P_n_, C_i_, T_r_ and L_s_ of *E. plantagineum* leaves, but had no significant effect on G_s_, T_r_ and chlorophyll fluorescence parameters. Delaying the sowing date caused a decrease in P_n_, L_s_, qP, ETR, ΦpsII and an increase in C_i_ of Blue Bedder. However, the rule of Mixed Bedding was not obvious. According to previous studies^30–33^, the decrease in C_i_ and increase in L_s_ meant that the decrease in stomatal conductance was the reason for the decrease in P_n_, while the increase in C_i_ and decrease in L_s_ indicated that the decrease in photosynthetic activity of mesophyll cells might be the reason for the decrease in P_n_. The sowing date mainly improved the P_n_ of Blue Bedder by improving the photosynthetic activities of mesophyll cells such as qP, ETR, and ΦpsII. Considering that the F_v_/F_m_ of Mixed Bedding was less than 0.8 at each sowing date, and its growth was subjected to external stress, the specific photosynthetic mechanism may be affected, and whether the effect of the specific sowing date on Mixed Bedding was the same as that of Blue Bedder needs to be further investigation.

The sowing date was an important factor influencing the yield formation of *E. plantagineum*. Król et al.^34^ had conducted planting trials in Poland and found that under the dense planting condition of 45 cm row spacing and 25 plants per square metre, the early sowing in April was conducive to increasing in the yield and 1000 grain weight of *E. plantagineum* in the region. However, in this study, except for the T1 sowing date of Mixed Bedding, both Mixed Bedding and Blue Bedder showed the increasing trend of delayed 1000-grain weight along with the sowing date, while the yield of Blue Bedder was the highest at the T1 sowing date, and the yield of Mixed Bedding was the highest at the T4 sowing date. This shows that density, sowing date and variety have certain effects on the yield characteristics of *E. plantagineum*. Correlation analysis showed that sowing date had a very significant positive correlation with total canopy PeR at the flowering stage and 1000 grain weight, and a very significant negative correlation with total canopy CaR at the flowering stage. Total canopy CaR and PeR at the flowering stage were significantly correlated with yield and photosynthetic traits (P_n_, C_i_, L_s_, F_o_, F_v_/F_m_, ETR, ΦpsII, SPAD, etc.). In addition, sowing date had a significant effect on total canopy CaR, PeR, P_n_, C_i_ and L_s_ of *E. plantagineum*, as well as on 1000 grain weight and yield. Therefore, different sowing dates can improve the photosynthetic capacity and thus the yield by adjusting the canopy structure of the population and the photothermal resources (Supplementary Information).

## Conclusion

The results showed that the photosynthetic, fluorescence and yield characteristics of the two *E. plantagineum* cultivars showed some differences. Under the experimental design conditions, the yield and photosynthetic performance of Blue Bedder were better than that of Mixed Bedding under the same sowing date. Sowing date had significant effects on the total canopy CaR, PeR, P_n_, C_i_ and L_s_, as well as on the 1000 grain weight and yield of *E. plantagineum*. Under the same cultivar conditions, with a delay in sowing date, the leaf SPAD, P_n_, T_r_, L_s_, qP, ETR, ΦpsII and yield of Blue Bedder decreased and reached a maximum at T1, whereas the SPAD, P_n_, T_r_, WUE, L_s_, F_o_, F_m_, qP, ETR, ΦpsII and yield of Mixed Bedding reached a maximum at T4. Therefore, different sowing dates can optimise the population structure and photothermal resources by adjusting the growth process to improve the photosynthetic capacity and thus increase the yield. The analysis showed that the sowing yield of Blue Bedder was the highest at T1 period and that of Mixed Bedding was the highest at T4 period. Therefore, cross sowing could improve the yield of *E. plantagineum*, and the sowing dates of the highest yield for different varieties were different.

### Supplementary Information

Supplementary Information.

## Data Availability

All data generated or analysed during this study are included in this published article.
